# Risk factors of hepatitis B virus infection among blood donors in Duhok city, Kurdistan Region, Iraq

**DOI:** 10.22088/cjim.9.1.22

**Published:** 2018

**Authors:** Nawfal R Hussein

**Affiliations:** 1 Department of Internal Medicine, College of Medicine, University of Duhok, Duhok, Kurdistan Region, Iraq.

**Keywords:** HBV, Iraq, Duhok, Risk factors

## Abstract

**Background:**

Hepatitis B virus (HBV) infection is a public health problem. The lack of information about the seroprevalence and risk factors is an obstacle for preventive public health plans to reduce the burden of viral hepatitis. Therefore, this study was conducted in Iraq, where no studies had been performed to determine the prevalence and risk factors of HBV infection.

**Methods:**

Blood samples were collected form 438 blood donors attending blood bank in Duhok city. Serum samples were tested for HBV core-antibodies (HBcAb) and HBV surface-antigen (HBsAg) by ELISA. Various risk factors were recorded and multivariate analysis was performed.

**Results:**

5/438 (1.14%) of the subjects were HBsAg positive (HBsAg and HBcAb positive) and 36/438 (8.2%) were HBcAb positive. Hence, 41 cases were exposed to HBV and data analysis was based on that. Univariate analysis showed that there were significant associations between history of illegitimate sexual contact, history of alcohol or history of dental surgeries and HBV exposure (p<0.05 for all). Then, multivariate analysis was conducted to find HBV exposure predictive factors. It was found that history of dental surgery was a predictive factor for exposure to the virus (P=0.03, OR: 2.397).

**Conclusions:**

This study suggested that the history of dental surgery was predictive for HBV transmission in Duhok city. Further population-based study is needed to determine HBV risk factors in the society and public health plan based on that should be considered.

Hepatitis B virus (HBV) is a public health problem and a major cause of chronic liver diseases. Approximately 350 to 400 million people are infected with such a virus with an estimate of one million lives are lost yearly due to the infection and its complications (1, 2). The prevalence of HBsAg positivity differs from a country to another which ranges from 0.5% in some developed countries to 8% in some Asian countries. In a previous study in Iran, a neighboring country to Iraq, it was shown that approximately 1% and around 5% of the general population were chronic HBsAg carriers and had previous exposure to HBV infection, respectively (3, 4). In the same study, they found age and marriage as risk factors for HBV (3). In another study conducted in Saudi Arabia recruiting blood donors, 3.0% of the samples were positive for HBsAg and 18.7% were positive for HBcAb (5). In a study conducted in Iraq recruiting 69915 blood donors, 0.2% of the donors were positive for HBsAg and 2.1% were positive for HBcAb (6). In previous studies from our region, the prevalence of HBV positivity was less than 1% (7-9). Unfortunately, the risk factors for HBV transmission were not studied thoroughly in Iraq. HBV can be prevented through pre exposure immunization targeting the risk group (10). 

In Iraq, HBV vaccination is a part of expanded program of vaccination targeting new born babies. All other at-risk groups are urged to take the vaccination, however, there is no systematic and scientific program of vaccination registration and surveillance. For the establishment of public health plan to combat HBV infection, studying the risk factors for transmission of infection is of great importance. It is known that the major risk factors for HBV transmission are blood and transfusion of blood products, pregnancy, healthcare workers, tattoo, drug abuse, and high risk sexual behaviors (10, 11). 

Since the distribution of HBV risk factors is important for any public health prevention plan, this cross-sectional study was conducted to determine the prevalence of HBV and associated risk factors in blood donors in Duhok City, Kurdistan region, northern Iraq. 

## Method

**Blood samples: **Blood samples were collected for 438 blood donors attending Duhok Blood transfusion center over a period of 4 months. A 5cc syringe and needle were used to bleed approximately 5 ml of blood from each donor. Then, the blood samples were centrifuged at 1500 rpm for 3 min to obtain serum. Samples were frozen in –20°C until the test were performed. All subjects were asked to fill in a self-filling administered questionnaire. The questionnaire covered marital status, history of blood transfusion, history of illegitimate sexual activities (multiple partners outside marriage), alcohol consumption, history of dental procedures, history of surgery, history of accidental injuries, history of drug use and HBV vaccination history.

**HBsAg and HBcAb ELISA:** HBsAg and HBcAb were studied by commercial LIAISON® XL diagnostic system (USA) following manufacturer’s instruction. First, specific monoclonal antibodies (anti HBsAg and anti HBcAb) were fixed to the surface of microwells. Sera from subjects were then added to the microwell and secondary conjugated monoclonal antibody, conjugated with horseradish peroxidase (HRP) were added. Unbound serum proteins and HRP conjugate were then washed off. The substrate was added after blocking the enzymatic reaction and optical density was measured by an ELISA reader. 

The study was approved by the College of Medicine Scientific and Ethics Committee, University of Duhok and Blood Bank, Duhok, Kurdistan region, Iraq. Written informed consent was obtained from all subjects before data collection.

**Statistics: **Chi-square test was applied to assess the associations between HBV positivity and different variables. All variables that achieved a p-value <0·2 were included in the multivariate study. Step-down regression was used where the variable with the lowest partial correlation was eliminated first. Variable confounder was determined if its deletion changed the coefficient by 20% of a statistically significant predictor. All computations were carried out using SPSS Version 21.

## Results

Blood samples were collected from 438 subjects and were examined for HBsAg and HBcAb. All the blood donors were males and the mean age of recruited subjects was 36.3±9.5 year. 365/438 (83.3%) of the subjects were married. In this study, 5/438 (1.14%) were HBsAg positive (HBsAg and HBcAb positive) and 36/438 (8.2%) were HBcAb positive. Hence, 41 cases were exposed to HBV (HBV positivity) and data analysis was based on that. Overall, 46/438 (10.5%) of the recruited subjects were vaccinated. Univariate analysis showed that 20/397 (5%) of the HBV-negative subjects gave a history of illegitimate sexual contact which was significantly lesser than that of HBV positive patients (7/41 (17%) P=0.06). Also, a significant association was found between history of alcohol and HBV exposure (P=0.03). 

Additionally, a significant association was found between history of dental surgeries and HBV positivity (P=0.011). Then, multivariate analysis was conducted to find HBV exposure predictive factors. It was found that history of dental surgery and illegitimate sex were predictive factors (table 1). 

The removal of history of drug use decreased the coefficient of illegitimate sex by 20% Then, to confirm the confounder effect of history of drug use upon the history of illegitimate sex, the data were stratified according to the history of drug use and the odd ratio was studied. It was found that there was different odds ratio (OR) for each group (for negative history of drug use OR=4.55, 95% CI: 1.48-13.87 and for positive history of drug use OR=2.61, 95% CI: 0.21-31.94) confirming the confounder effect. 

**Table 1:** Risk factors associated with HBV positivity

**Risk factors**		**HBV** **positive**	**HBV** **negative**	**Univariate analysis**		**Multivariate analysis**	
				**P-value**	**Crude OR**	**P-value**	**Adjusted OR**
Marital status	Single	6	76	0.312	0.7	0.3	0.7
	married	35	321				
History of receiving blood	yes	9	58	0.079	1.64	0.23	1.65
	No	32	339				
History of street drug use	yes	3	56	0.103	0.48	0.1	0.3
	No	38	341				
History of dental surgery	Yes	33	251	0.011	2.4	0.03	2.3
	No	8	146				
History of surgical operation	Yes	14	155	0.113	0.81	0.68	0.68
	No	27	242				
History of tattooing	Yes	8	51	0.088	1.64	0.66	1.23
	No	33	346				
History of accidental injury	yes	4	32	0.204	1.23	0.64	0.72
	No	37	365				
History of alcohol	Yes	14	72	0.03	2.34	0.5	1.14
	No	27	325				
History of illegitimate sex	yes	7	20	0.006	3.88	0.018	3.3
	No	34	377				

## Discussion

HBV is a common public health problem especially in developing countries and is associated with serious consequences such as liver cirrhosis and hepatocellular carcinoma. In the Middle East, the prevalence of HBV ranges around 3% in Iraq, Iran and Syria to about 7% in Yemen and some regions in Saudi Arabia (3, 5, 12). The prevalence of HBV was studied thoroughly in Iraq. The prevalence varied in this country according to the geographical regions. In studies from the middle part of Iraq investigating the prevalence of HBsAg positivity in the cities of Babylon, the seroprevalence was found to be approximately 0.7%. The same results were found in Kurdistan region, Northern Iraq (12-15). However, in a study conducted in Kerbala, southern Iraq, the prevalence rate was 3.5% (12). 

In this study, it was shown that 1.14% of the blood donors were HBsAg positive and 8.2% of the recruited subjects were HBcAb positive indicating old resolved infection. The total population of Duhok City is about 2,277,151, therefore it is estimated that 25,959 chronic carriers live in this city and 186,726 have resolved HBV infection. This number of chronically infected subjects may pose a threat upon the society because of the possibility of the infection transmission. Additionally, treating the infection and its complications in this large number of subjects represents a heavy burden on the national economy. Therefore, an urgent public health plan is needed for surveillance and infection prevention. HBV can be transmitted vertically from mother to new born baby during delivery. Besides, it can be transmitted sexually, through blood and blood products transfusion and exposure to contaminated blood via needles (10). There is a variation in the mode of transmission of the virus from a community to another in accordance with norms, traditions and social factors. In a study conducted in China recruiting more than 8000 subjects, it was found that male gender, old age and history of surgical operations were associated with high risk of HBV positivity (16). In another study conducted in Iran, marriage and old age were associated with high risk of acquiring HBV infection (3). In Turkey, dialysis, family history of HBV and sexual contact with HBV positive subjects were found as the risk factors for acquiring HBV infection (17-19). No studies have been conducted to determine the risk factor of HBV infection in Iraq. In this study, dental surgery was found as a predictive factor for HBV transmission in the community. This study is of exceptional importance for public health planner in Iraq because to the best of our knowledge, it is the first project studying the predictive factors of HBV infection in the country. Illegitimate sex was also associated with the high prevalence of HBV exposure, though the effect of drug use as a confounder could not be excluded. Hence, planning of prevention public health program should concentrate upon education regarding safe and protective sex. In addition, monitoring dental clinics for safe practice and infection control measures may help decrease the transmission of such an infection. Blood transfusion and surgery were not predictive factors for HBV infection. This might return back to the strict policy of testing blood and blood products and strict infection control measures in the operation theatre in Duhok City. HBV infection can be prevented by vaccination. The HBV vaccination coverage rate varies from a country to another with an estimated coverage rate between 24% in some African countries to more than 70% in western countries (10, 20). In a previous study in Iraq, it was shown that all the recruited subjects, who were healthcare workers, were vaccinated (8). In this study, the vaccination rate was only 10.5%, despite the education campaigns encouraging vaccination. Therefore; a better plan is required to vaccinate blood donors and more efforts are needed to convince blood donors of the importance of vaccination. 

Our study has limitations. First, the sample size was relatively small. Nonetheless, this is more likely to hide true-positive associations rather than to produce false-positive results. Second, the prevalence of HBV in blood donors may not reflect the prevalence of infection in the society. Nevertheless, this does not negate the importance of the project findings of potential risk factor in the society. 

To conclude, 8.2% of general population in this province showed prior exposure to HBV and 1.14% were HBsAg carriers. This report suggested that a previous history dental procedure was predictive for the transmission of HBV in our region. Furthermore, population-based study is needed to determine HBV risk factors in the society and public health plan rooted on that should be considered.
